# Detection of occult carcinomatous diffusion in lymph nodes from head and neck squamous cell carcinoma using real-time RT–PCR detection of cytokeratin 19 mRNA

**DOI:** 10.1038/sj.bjc.6603073

**Published:** 2006-04-18

**Authors:** L Tao, M Lefèvre, S Ricci, P Saintigny, P Callard, S Périé, R Lacave, J-F Bernaudin, J Lacau St Guily

**Affiliations:** 1 Service d'ORL et Chirurgie Cervico-Faciale, Université Pierre et Marie Curie, Paris VI, Hôpital Tenon APHP, 4 rue de la Chine, Paris 75020, France; 2 Anatomie-Pathologique, EA 3499 Université Pierre et Marie Curie Paris VI, Hôpital Tenon APHP, Paris 75020, France; 3 Histologie-Biologie Tumorale, EA 3499 Université Pierre et Marie Curie Paris VI, Hôpital Tenon APHP, Paris 75020, France; 4 Department of Otolaryngology-HNS, Eye and ENT Hospital of Fudan University, Shanghai, China

**Keywords:** head and neck squamous cell carcinoma, cytokeratin 19, real-time RT–PCR, lymph nodes, micrometastasis, neck dissection

## Abstract

The aim of the present study was to evaluate the occult lymph node carcinomatous diffusion in head and neck squamous cell carcinoma (HNSCC). A total of 1328 lymph nodes from 31 patients treated between 2004 and 2005 were prospectively evaluated by routine haematoxylin–eosin–safran (HES) staining, immunohistochemistry (IHC) and real-time Taqman reverse–transcriptase polymerase chain reaction (real-time RT–PCR) assay. Amplification of cytokeratin 19 (CK19) mRNA transcripts using real-time RT–PCR was used to quantify cervical micrometastatic burden. The cervical lymph node metastatic rates determined by routine HES staining and real-time RT–PCR assay were 16.3 and 36.0%, respectively (*P*<0.0001). A potential change in the nodal status was observed in 13 (42.0%) of the 31 patients and an atypical pattern of lymphatic spread was identified in four patients (12.9%). Moreover, CK19 mRNA expression values in histologically positive lymph nodes were significantly higher than those observed in histologically negative lymph nodes (*P*<0.0001). These results indicate that real-time RT–PCR assay for the detection of CK19 mRNA is a sensitive and reliable method for the detection of carcinomatous cells in lymph nodes. This type of method could be used to reassess lymph node status according to occult lymphatic spread in patients with HNSCC.

The presence of metastatic cervical lymph nodes is a major prognostic factor in patients with head and neck squamous cell carcinoma (HNSCC) (Shores *et al*, 2004). Staging of cervical lymph nodes, therefore critical, is usually limited to routine histologic examination of only a few hematoxylin–eosin (HE)-stained sections of each node (Barrera *et al*, 2003). Various reports have demonstrated that such a routine analysis may fail to detect a significant number of nodal metastases in lung and breast cancers (International (Ludwig) Breast Cancer Study Group, 1990; Wu *et al*, 2001). Regional recurrence rates of approximately 10% are reported in patients with histologically negative neck dissection specimens, suggesting that carcinomatous cells were present but not detected in the resected nodes (Tu, 1999). In a recent report, approximately 5–20% of HNSCC patients harboured occult metastases not identified by routine histopathologic examination but detected by other methods such as immunohistochemistry (IHC) or molecular biology, that is, reverse transcriptase–polymerase chain reaction (RT–PCR) (Hamakawa *et al*, 2000a; Godfrey *et al*, 2001; Shores *et al*, 2004; Le Pimpec-Barthes *et al*, 2005).

Additional use of a molecular investigation, such as RT–PCR, has been largely developed in various solid tumours to determine the incidence of micrometastases missed during routine light microscopy (Ambrosch and Brinck, 1996). However, some of these molecular studies performed in HNSCC have been suspected to have a low specificity (Kawamata *et al*, 1999; Hamakawa *et al*, 2000a). We therefore decided to design a method based on a real-time Taqman PCR reverse–transcriptase polymerase chain reaction (real-time RT–PCR) assay to detect cytokeratin 19 (CK19) mRNA marker of occult tumour cells in lymph nodes of patients undergoing neck dissection for HNSCC as such a marker has been shown to detect accurately micrometastases in patients with lung, oesophageal, gastric, breast or colorectal carcinoma (Izbicki *et al*, 1997; Czerniecki *et al*, 1999; Weitz *et al*, 1999; Gai *et al*, 2000; Saintigny *et al*, 2005). Moreover CK19 has been demonstrated to be a component of the cell cytoskeleton of HNSCC (Cohen-Kerem *et al*, 2002; Welkoborsky *et al*, 2003) and can be considered to be an appropriate marker of HNSCC carcinomatous cells.

The aim of this prospective study was therefore to develop a quantitative CK19 mRNA RT–PCR assay for the detection and quantification of occult micrometastases in cervical lymph nodes collected during systematic neck dissection of HNSCC. We hypothesised that the detection of carcinomatous lymph node involvement using more sensitive methods than routine histopathologic examination, such as real-time RT–PCR, could change the nodal status, as well as the patient's subsequent treatment regimens (Shores *et al*, 2004).

## MATERIALS AND METHODS

### Patients and neck dissection samples

This study, conducted according to the institutional and ethical rules concerning research on tissue specimens, was exempted from informed consent from the patients (CCPPRB Paris Pitié Salpétrière). A prospective series of 31 patients with primary squamous cell carcinoma of the oral cavity (*n*=7, 22.6%), oropharynx (*n*=6, 19.4%), hypopharynx (*n*=17, 54.8%) and larynx (*n*=1, 3.2%) who underwent surgical treatment including unilateral or bilateral selective neck dissection between 2004 and 2005 at the Department of Otolaryngology Head and Neck Surgery of Tenon hospital (Paris University VI) were entered in the present study (Table 1). The average age of these patients was 59.5±9.3 year (range: 40–75 years). In these 31 patients, a total of 42 selective neck dissections (11 patients had bilateral dissections) were performed, and 1328 lymph nodes (mean: 42.8±20.6 nodes per patient) were obtained from the neck dissection samples. These 1328 lymph nodes were collected from 178 different groups distributed at various levels according to the topographic classification of neck nodes by Robbins *et al* (2002).

Lymph nodes, macroscopically isolated, were cut into 3 mm slices. The even-numbered slices were formalin-fixed (24-h in 20% buffered-formalin) and paraffin-embedded for routine HE–safran (HES) staining, and the odd-numbered slices were frozen in liquid nitrogen and stored at −80°C until RNA extraction. The entire procedure after neck dissection to freezing was performed within 1 h.

### Control lymph nodes and peripheral blood mononuclear cells (PBMNs)

In all, 17 lymph nodes were collected from patients undergoing sigmoidectomy for sigmoid diverticulitis (*n*=6) or elective carotid endarterectomy (*n*=11). None of these patients had a history or clinical evidence of cancer. Five millilitres of blood from 29 healthy volunteers were collected by venipuncture. Peripheral blood mononuclear cells were isolated by density centrifugation on Ficoll gradient (catalog number U04; Unisep+Novamed, Israel). Control lymph nodes and blood mononuclear cells were processed for RNA extraction.

### Real-time RT–PCR assay

Frozen specimens from neck dissection lymph nodes, CAPAN-1 cell line (human pancreas adenocarcinoma cells) and PBMNs were stored at −80°C until RNA extraction. CAPAN-1 cell line, obtained from the American Tissue Culture Collection (ATCC, Rockville, MD, USA), was selected to standardise the CK19 mRNA marker (Saintigny *et al*, 2005). The odd-numbered frozen sections of lymph nodes from the same level according to the above-mentioned topographic classification were pooled as a single specimen for RNA extraction. Total RNA was extracted using the Trizol reagent (Life Technology, Gaithersburg, MD, USA) and purified according to the manufacturer's instructions. The quantity and quality of the extracted RNA were confirmed by absorption spectrophotometry at 260 and 280 nm. cDNA was reverse-transcribed from 2 *μ*g of total RNA in a 20 *μ*l reaction mix using the Moloney-Murine Leukemia Virus Superscript II RNase H-Reverse Transcriptase Kit from Gibco-BRL (Invitrogen, Cergy-Pontoise, France) according to the manufacturer's instructions.

For CK19 mRNA detection, primers (Proligo Sigma, Paris, France) and probe (MWG Biotechnology Ebersberg, Germany) were designed to avoid the detection of the two known pseudogenes (Rund *et al*, 1999; Van Trappen *et al*, 2001), and a custom-labelled fluorescence probe was synthesised with FAM (6-carboxyfluorescein) and TAMRA (6-carboxyltetramethylrhodamine). CK19 (75 bp) – forward primer: 5-TCG ACA ACG CCC GTC TG-3, reverse primer: 5-CCA CGC TCA TGC GCA G-3, probe: 5-(FAM)-CCG AAC CAA GTT TGA GAC GGA ACA GG-(TAMRA)-3. We evaluated the use of human leucocyte class IC (HLAIC) as an internal control (Rasmussen, 2001). Primers (Proligo Sigma, Paris, France) for HLAIC mRNA detection and HLAIC probe (MWG Biotechnology Ebersberg, Germany) were designed using Perkin-Elmer Primer Express software with Proligo (Paris, France), and a custom-labelled fluorescence probe was synthesised with HEX (hexachloro-6-carboxyfluorescein) and TAMRA. Human leucocyte class IC (63 bp) – forward primer: 5-GGG TAT GAC CAG TCC GCC T-3, reverse primer: 5-GGA GCG CAG GTC CTC GTT-3, probe: 5-(HEX)-CGA CGG CAA GGA TTA CAT CGC CCT-(TAMRA)-3. The specificities of primers and probe sequences were confirmed by BLAST analysis (www.ncbi.nlm.nih.gov/BLAST/).

Real-time RT–PCR assays were carried out using a 25 *μ*l reaction mix on the iCycler iQ (Bio-Rad, Hercules, CA, USA) Taqman chemistry system including 5 *μ*l of 20-fold diluted total reverse transcription reaction mix, 300 nM of forward primer, reverse primer and 200nM of probe, 12.5 *μ*l of Taqman PCR Master Mix (Applied Biosystems, Foster City, CA, USA) and sterile water. Thermocycler conditions were 10 min at 95°C before 40 cycles at 95°C for 15 s and at 60°C for 60 s (two-step reaction). Each amplification was performed in triplicate, with a negative control (absence of cDNA) and a positive control (cDNA of the standard for the mRNA tested). The parameter threshold cycle (*C*_t_) is defined as the fractional cycle number at which the fluorescence generated by cleavage of the probe exceeds a fixed threshold above baseline. Lymph node levels were considered to be positive when all triplicates were positive for CK19 mRNA and when the internal control was amplified within the usual range.

**Figure 1:**
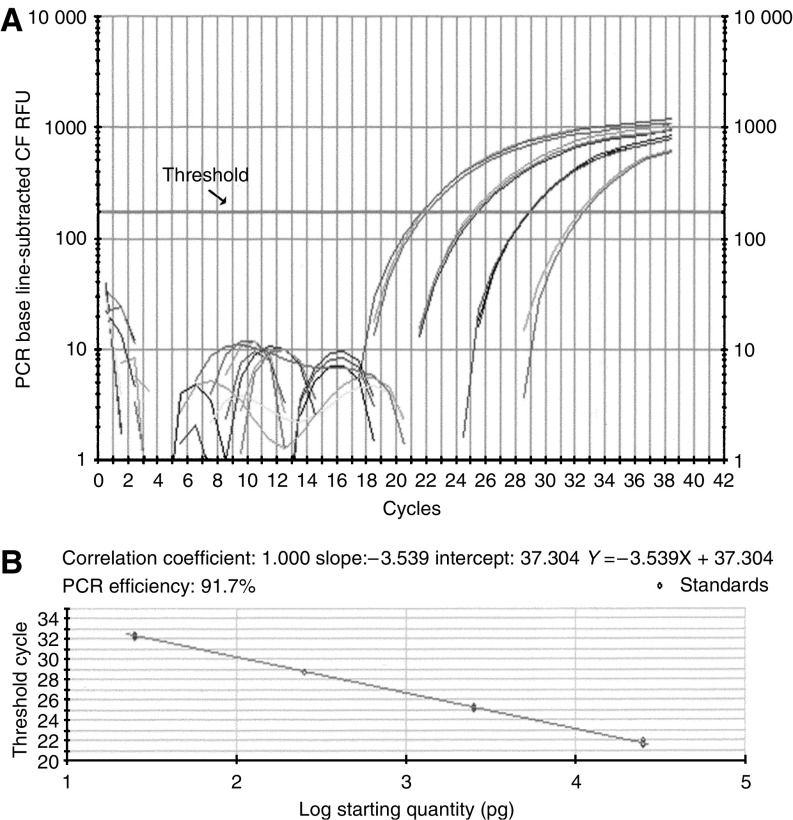
Amplification of CK19 mRNA by real-time RT–PCR assay (iCycler iQ). (**A**) Amplification plots of four 10-fold serial dilutions of the standard CK19 mRNA from CAPAN-1 cells total RNA. The initial amount of total mRNA is displayed: 2.5 × 10^4^, 2.5 × 10^3^, 2.5 × 10^2^ and 25 pg. Threshold cycle (*C*_t_) is calculated as the cycle at which fluorescence signal exceeds a fixed threshold line. (**B**) Standard curve of real-time RT–PCR CK19 assay. *C*_t_ is plotted against the starting quantity of CAPAN-1 cell total RNA.

The standard curve was plotted for each reaction using four 10-fold serial dilutions of standard cDNA, and the amplification efficiency (AE) was calculated using Bio-Rad software (Bio-Rad, Hercules, CA, USA) according to the following equation: AE=10^−1/*m*^, where *m* is the slope of the line determined by the four dilutions of standard cDNA for the marker tested. The PCR reaction was linear from the first dilution to the fourth dilution of all standards (Figure 1). The best standard was defined by the closest expression level between the mRNA marker and the internal control and by an AE for the CK19 mRNA marker and internal control greater than 90%. Quantitation of mRNA relative to the standard level of expression of the genes of interest was defined as a relative quantity (RQ) and was given by the following equation (Livak and Schnittgen, 2001): 
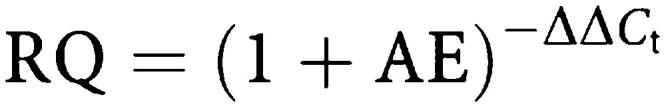
, where ΔΔ*C*_t_==((*C*_t gene of interest_−*C*_t internal control_)_sample_−(*C*_t gene of interest_−*C*_t internal control_)_standard_).

### Immunohistochemistry

Immunohistochemistry was performed on 4 μm sections of formalin-fixed, paraffin-embedded samples from 203 lymph nodes, negative after HES staining and in which CK19 mRNA was detected by real-time RT–PCR assay and, as controls, on five HES-positive lymph nodes. Immunostaining was carried out using the Ventana immunostainer automatic protocol (Saintigny *et al*, 2005) with pancytokeratin antibody (AE1/AE3, Dako Cytomation M3515, diluted to 1 : 100).

### Statistical analysis

Categorical variables were compared using the *χ*^2^ test. Continuous variables were compared using the Mann–Whitney *U*-test. All *P*-values were two-sided. Differences were considered significant for *P*<0.05. These statistical analyses were performed using SPSS version 11.0 software.

## RESULTS

### Histopathologic examination

Metastatic carcinomatous cells were observed after HES staining in 33 (2.5%) of the 1328 lymph nodes. The frequencies of nodal metastases were 2.0% (six of 301) in the oral cavity, 0.9% (two of 226) in the oropharynx, 3.3% (25 of 755) in the hypopharynx and 0% (zero of 46) in the larynx (Table 1). Of the 178 cervical lymph node levels, 29 (16.3%) investigated were considered to be metastatic. The details of these results are presented in Table 2.

### Real-time RT–PCR assay

No amplification product was observed when no template was added. The amplified products obtained from RNA extracted from CAPAN-1 cell line for CK19 and HLAIC show the expected bands (75 and 63 bp) after agarose gel electrophoresis (data not shown). CK19 mRNA was not detected in either PBMN samples from healthy subjects or in RNA extracted from the 17 lymph nodes collected from patients without cancer (data not shown). Human leukocyte class IC expressed in histopathologically negative lymph nodes (*C*_t_=24.78±1.05) close to the levels observed in histopathologically positive lymph nodes (*C*_t_=24.35±1.25) has been demonstrated to be a particularly accurate internal control (*P*>0.05).

CK19 mRNA was detected in 236 lymph nodes from 64 lymph node levels; 203 of these lymph nodes were negative after histopathology examination. Detailed results of CK19 mRNA detection by real-time RT–PCR according to tumour site are shown in Table 2. Real-time RT–PCR gave a positive signal in 36.0% of all lymph node levels, while histopathology detected carcinomatous cells in 16.3% of lymph node levels. On the basis of these results, molecular analysis using real-time RT–PCR for CK19 mRNA detection gave significantly different results from routine histopathologic examination (*P*<0.0001).

Individual results per patient are shown in Table 3 for patients who underwent unilateral neck dissection and in Table 4 for patients who underwent bilateral neck dissections. Patients were staged as pN according to the usual histopathologic criteria (Hermanek *et al*, 1998) and mN according to CK19 mRNA real-time RT–PCR results. The nodal status of 13 (45.2%) of 31 patients (eight with unilateral neck dissection, five with bilateral neck dissections) would therefore have been changed if the molecular results were taken into account. Moreover, in nine of these 13 patients (five with unilateral neck dissection, four with bilateral neck dissections), nodal status would have been upstaged from pN0 to mN1, mN2b or mN2c, respectively (Hermanek *et al*, 1998).

In the 16 patients with lymph nodes metastases, analysis of the pattern of carcinomatous spread within lymph node levels showed that nodes from the IIa and IIb levels were free of metastatic cells on pathologic examination in five (16%) of them: CK19 mRNA was detected, while HES was negative in IIa and IIb lymph node levels in two of those patients.

**Figure 2:**
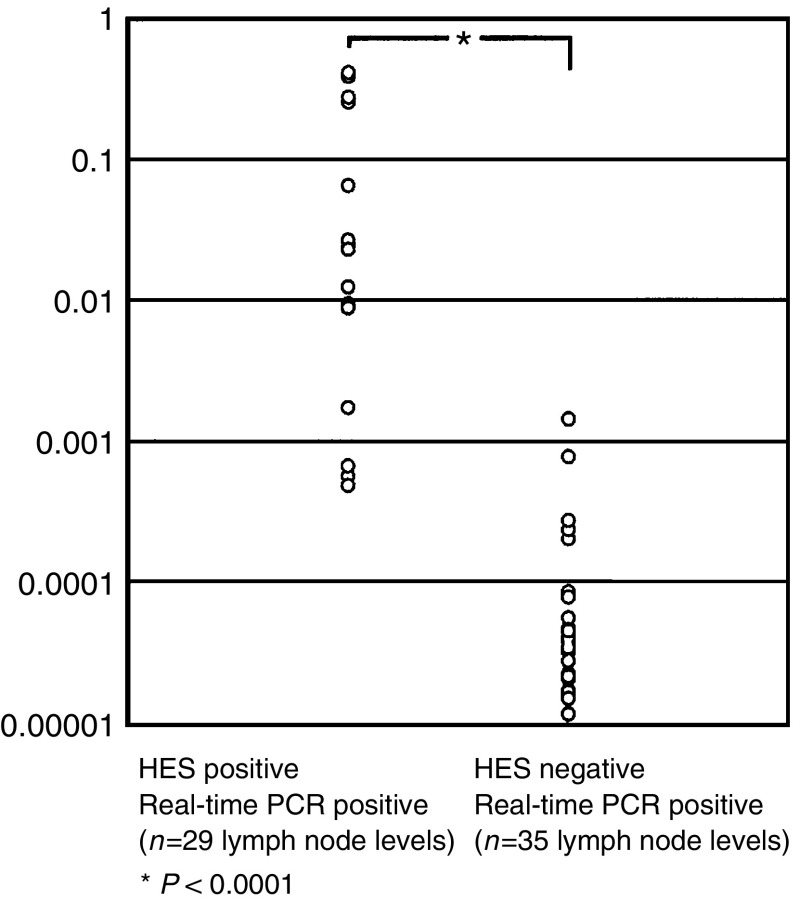
RQ (relative quantification of CKmRNA copies) according to the equation: 
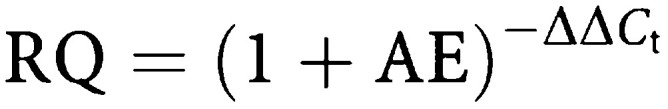
 of CK19 mRNA expression in 64 lymph node levels (29 histopathologically positive; 35 histopathologically negative).

In the 64 lymph node levels showing CK19 mRNA expression, the number of CK19 mRNA copies detected by real-time RT–PCR was evaluated by the RQ calculation as shown in Figure 2. The expression values of all histopathologically positive lymph node levels ranged from 4.85 × 10^−4^ to 4.15 × 10^−1^, values that were significantly higher than those for histologically negative lymph node levels, which ranged from 1.13 × 10^−5^ to 1.42 × 10^−3^ (*P*<0.0001).

### Immunohistochemistry

**Figure 3:**
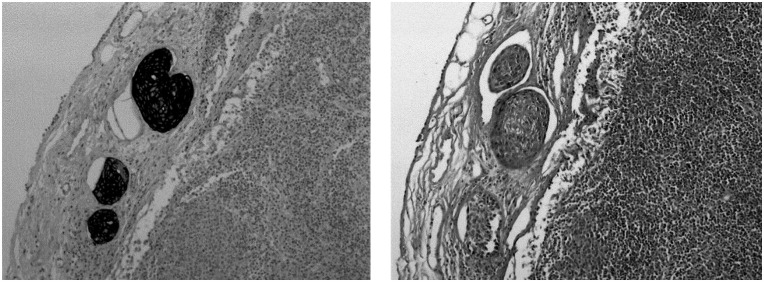
Immunohistochemistry on lymph nodes containing carcinomatous cells detected after HES staining (right) after incubation with an anticytokeratin monoclonal antibody (left) (final magnification × 400).

In the 203 lymph nodes that were histopathologically negative but CK19 mRNA positive on real-time RT–PCR, no carcinomatous cell was immunodetected by IHC, while in all the five HES-positive lymph nodes, carcinomatous cells were immunolabelled (Figure 3).

### DISCUSSION

Carcinomatous cell diffusion in cervical lymph nodes is a major determinant of therapy and prognosis for patients with HNSCC (Shores *et al*, 2004) as cure rates for patients with pathologically metastatic lymph nodes drop to one-half of those of patients without nodal involvement (Shah, 1990). As histopathologic analysis of neck dissection specimens is usually performed on several 3–4 mm sections from each lymph node, and as micrometastases represent tumour deposits measuring less than 2 mm in diameter, they can be easily missed on routine light microscopy (Hermanek *et al*, 1999; Genden *et al*, 2003). For example, it has been reported that 21.9% of patients with cancer of the oral cavity have micrometastases with an average diameter of 1.36 mm (Hamakawa *et al*, 2000b).

Various molecular markers, as mRNA expressed in tumour cells, particularly CK mRNAs, have therefore been investigated in order to detect the presence of occult tumour cells in lymph nodes (Shores *et al*, 2004). Previous studies using such markers in HNSCC have suggested a low specificity (Kawamata *et al*, 1999; Hamakawa *et al*, 2000a), particularly because of false-positive results associated with benign epithelial rests in lymph nodes found in 1.6% of cervical nodes, but only in lymph nodes from level I (Gnepp, 2000; Hamakawa *et al*, 2000b). The assay used in the present study, that is, RT–PCR detection of CK19 mRNA, has been developed in our laboratory for the detection of occult carcinomatous cells in mediastinal lymph nodes in non-small-cell lung carcinoma (Saintigny *et al*, 2005) and by other teams in various carcinomas (Van Trappen *et al*, 2001; Inokuchi *et al*, 2003; Favia *et al*, 2004). The primers and probe for CK19 mRNA detection were designed to avoid the detection of the two known pseudogenes (Rund *et al*, 1999; Van Trappen *et al*, 2001; Saintigny *et al*, 2005) and we used real-time RT–PCR with the Taqman chemistry. All blood cell samples from healthy donors and lymph nodes specimens collected from patients without cancer were negative, while CK19 mRNA was detected in all samples from the 29 lymph node levels shown to be metastatic on histopathology. We therefore consider that CK19 mRNA is a reliable marker for carcinomatous cell detection in HNCC as in other carcinomas (Van Trappen *et al*, 2001; Saintigny *et al*, 2005).

In this study, CK19 mRNA was detected in 35 of the 178 lymph node levels that were free of tumour cells on histopathology. Moreover, quantitative evaluation by the RQ of the amount of CK19 mRNA clearly showed a significant difference between histopathologically positive and negative lymph nodes. In a previous study, using dilution of CAPAN-1 cells in blood cells, we showed that the threshold of this method can be considered to detect one tumour cell within 10^4^ PBMNS (Saintigny *et al*, 2005).

These data suggest that real-time RT–PCR could potentially increase the sensitivity of routine pathological examination (Becker *et al*, 2004; Ferris *et al*, 2005), and could therefore be used routinely to examine all nodes removed during neck dissection that are negative on standard histopathologic examination.

Clinically, although the prognostic significance of micrometastases has not been established in HNSCC, a previous study suggested that the presence of occult micrometastases increases the risk of regional recurrence (Rhee *et al*, 2002). According to Ferlito *et al* (2000), selective neck dissection in HNSCC provides important information for prognostic purposes and therapeutic decisions. In the present study, occult micrometastases were detected in 13 out of 31 patients, and if these findings were taken into account, they would have changed the patients' nodal status and nine pN0 patients would have been mN-upstaged.

According to the work of Rouvière (1938), the lymphatic drainage of the head and neck region follows a relatively constant and sequential route. Level II is the first node level in the majority of HNSCC, but other levels are also likely to be the first node level, especially in tumours of the oral cavity (level I) or hypopharynx (level III) (Schauer *et al*, 2004). As a variable lymphatic drainage in HNSCC can be observed, tumour cells may be missed in unsuspected groups of lymph nodes (Byers *et al*, 1997). In our study, this variability of lymphatic drainage was observed in five out of 31 patients. In two of these patients, CK19 mRNA was detected in IIa and IIb lymph node levels that were negative on histopathology. In support of this idea, Mozzillo *et al* (2001) recently noted a 18% incidence of sentinel nodes outside the expected nodal drainage pattern, and Byers *et al* (1997) found that 15.8% of 277 patients with squamous cell carcinoma of the oral tongue had skip metastases.

Serial sectioning and IHC have been extensively developed for the detection of occult metastasis in HNCC and in a wide range of solid tumours (Ambrosch and Brinck, 1996). This method has the advantage of preserving cellular and tissue morphological features, but is expensive and time-consuming, and therefore cannot be used for most routine applications (Becker *et al*, 2004). However, it has been demonstrated that it does not allow a significantly higher detection rate than histopathologic examination of HE-stained sections (Woolgar, 1999; Hamakawa *et al*, 2000b). In our study, immunodetection of carcinomatous cells of the 203 nodes that were HES negative and real-time RT–PCR positive was negative. However, the clinical relevance of this detection, by real-time RT–PCR only, with respect to prognosis and treatment needs to be investigated by further clinical studies.

In conclusion, these results indicate that real-time RT–PCR assay for the detection of CK19 mRNA is a sensitive and reliable method for the detection of carcinomatous cells in lymph nodes in patients with HNSCC. This type of method could be used to reassess lymph node status according to occult lymphatic spread in patients with HNSCC.

## Figures and Tables

**Table 1:** Characteristics of patients with HNSCC

**Patients**	**Oral cavity**	**Oropharynx**	**Hypopharynx**	**Larynx**
Number	7	6	17	1
Age	55.7±5.7	69.0±5.5	57.4±9.7	64

*Gender*				
Male	5	5	14	0
Female	2	1	3	1

*T stage*				
T1–2	6	6	10	0
T3–4	1	0	7	1

*N stage*				
N0	3	3	8	1
N1–3	4	3	9	0

*Lymph nodes*				
Total (%)^a^	301 (22.7%)	226 (17.0%)	755 (56.9%)	46 (3.4%)
Positive (HES)^b^	6 (2.0%)	2 (0.9%)	25 (3.3%)	0 (0%)

HES=haematoxylin–eosin–safran; HNSCC=head and neck squamous cell carcinoma.

a % of all lymph nodes collected.

b After usual histopathologic examination.

**Table 2:** Results of histopathologic examination and CK19 mRNA real-time RT–PCR in the various cervical lymph node levels expressed as the number of positive levels/total number of levels investigated

	**Oral cavity**		**Oropharynx**		**Hypopharynx**		**Larynx**		**All tumours**	
**Lymph node levels^a^**	**HES**	**RT–PCR**	**HES**	**RT–PCR**	**HES**	**RT–PCR**	**HES**	**RT–PCR**	**HES**	**RT–PCR**
Level I	2/10	4/10	0/2	0/2	0/0	0/0	0/0	0/0	2/12	4/12
Level IIa	1/9	5/9	1/5	1/5	9/24	12/24	0/2	0/2	12/40	18/40
Level IIb	0/9	7/9	0/7	2/7	5/23	9/23	0/2	0/2	6/41	18/41
Level III	0/9	2/9	0/7	1/7	6/24	8/24	0/2	0/2	6/42	11/42
Level IV	0/7	2/7	0/7	2/7	1/23	7/23	0/2	0/2	1/39	11/39
Level V	0/0	0/0	1/1	1/1	1/2	1/2	0/0	0/0	2/3	2/3
Level VI	0/0	0/0	0/0	0/0	0/1	0/1	0/0	0/0	0/1	0/1

Total	5/44	20/44	2/29	7/29	22/97	37/97	0/8	0/8	29/178	64/178
%	11.4	45.5	6.9	24.1	22.7	38.1	0	0	16.3^b^	36.0^b^

CK19=cytokeratin 19; HES=haematoxylin–eosin–safran; RT–PCR=reverse transcriptase–polymerase chain reaction.

a Neck dissection samples have been categorised according to the topographic classification of cervical lymph node levels proposed by Robbins *et al* (2002).

b Difference between HES and real-time RT–PCR results, *P*<0.0001.

**Table 3:** Results of histopathologic and molecular node status in patients with unilateral neck dissection

				**Lymph node levels^c^**	
**Patients age/gender**	**Tumour sites**	**pN^a^**	**mN^b^**	**Histopathology^a^**	**PCR^b^**
50/M	Oral cavity	0	2b^d^	—	Ia, IIb
57/M	Hypopharynx	0	2b^d^	—	IIa, IIb
75/M	Oropharynx	0	0	—	—
69/M	Oropharynx	0	0	—	—
52/M	Hypopharynx	2	2a	IIa	IIa
58/M	Hypopharynx	0	0	—	—
73/M	Oropharynx	0	2b^d^	—	IIb, IV
74/M	Hypopharynx	2b	2b	IIa, IIb	IIa, IIb
69/M	Hypopharynx	2b	2b	III	III
50/F	Oral cavity	1	2b^d^	IIa	IIa, III
50/M	Hypopharynx	1	1	IIa	IIa
64/F	Oropharynx	0	0	—	—
54/M	Hypopharynx	0	0	—	—
41/M	Hypopharynx	1	2b^d^	III	IIa, IIb, III, IV
72/M	Oropharynx	1	2b^d^	V	IV, V
51/M	Oral cavity	2b	2b	Ib	Ia, Ib, IIa, IIb
69/M	Hypopharynx	0	0	—	—
55/M	Hypopharynx	2b	2b	IIa, III	IIa, III
64/M	Oral cavity	0	1^d^	—	IIb
61/M	Oral cavity	0	2b^d^	—	IIa, IIb

RT–PCR=reverse transcriptase–polymerase chain reaction.

a Pathologic lymph node staging according to histopathologic examination; —indicates the absence of detected carcinomatous cells.

b Molecular lymph node staging according to real-time RT–PCR detection of CK19 mRNA.

c Neck dissection samples have been categorised according to the topographic classification of the cervical lymph node levels proposed by Robbins *et al* (2002).

d Change in nodal status according to real-time RT–PCR CK19 results.

**Table 4:** Results of histopathologic and molecular node status in patients with bilateral neck dissections

				**Lymph node levels – R^c^**	**Lymph node levels – L^c^**		
**Patients age/gender**	**Tumour sites**	**pN^a^**	**mN^b^**	**Histopathology^a^**	**PCR^b^**	**Histopathology^a^**	**PCR^b^**
61/M	Oropharynx	2a	2b^d^	IIa	IIa, IIb, III	—	—
66/F	Hypopharynx	2c	2c	IIa, IIb, III	IIa, IIb, III	IIa	IIa, III, IV
68/M	Hypopharynx	2b	2b	IV, V	IV, V	—	—
63/M	Hypopharynx	0	2c^d^	—	III, IV	—	IV
53/M	Hypopharynx	2c	2c	IIa	IIa, IIb	III	III
64/F	Larynx	0	0	—	—	—	—
40/M	Hypopharynx	2c	2c	IIb, III	IIb, III	IIa, IIb	IIa, IIb
55/M	Oral Cavity	2b	2b	—	—	Ib, IIa, IIb	Ib, IIa, IIb
59/F	Oral Cavity	0	2c^d^	—	IIa, IIb, IV	—	IIb, III, IV
52/F	Hypopharynx	0	2c^d^	—	IIa, IV	—	IIa, IIb, IV
55/F	Hypopharynx	0	1^d^	—	—	—	IIb

RT–PCR=reverse transcriptase–polymerase chain reaction.

a Pathologic lymph node staging according to histopathologic examination; —indicates the absence of detected carcinomatous cells.

b Molecular lymph node staging according to detection of CK19 mRNA by real-time RT–PCR.

c Neck dissection samples have been categorised prospectively according to the topographic classification of cervical lymph node levels proposed by Robbins *et al* (2002) (R: right; L: left).

d Change in nodal status according to real-time RT–PCR CK19 results.
